# Development and internal validation of a nomogram for predicting the severity of community-acquired pneumonia in children

**DOI:** 10.3389/fcimb.2026.1797095

**Published:** 2026-06-19

**Authors:** Min Wang, Juan Wang, Ping Liu, Chong Hu, Yanzi Zhang, Xin Lv, Lingdong Zhu

**Affiliations:** 1 Clinical Laboratory, Children’s Hospital Affiliated to Shandong University, Jinan, China; 2 Clinical Laboratory, Jinan Children’s Hospital, Jinan, China

**Keywords:** community acquired pneumonia, early prediction model, etiology, inflammatory marker, nomogram

## Abstract

**Objective:**

To investigate the etiological spectrum, epidemiological characteristics of community-acquired pneumonia (CAP) in hospitalized children, identify risk factors for severe CAP (sCAP) and establish a predictive nomogram model.

**Methods:**

A retrospective study included 1486 children with CAP admitted to Jinan Children’s Hospital from January 2023 to December 2024. Etiological and epidemiological features were analyzed by age and season. Univariate and binary multivariate logistic regression were used to screen risk factors for sCAP. The nomogram model was constructed, with discriminative ability and calibration evaluated by receiver operating characteristic (ROC) and calibration curves.

**Results:**

The top detected bacteria were Streptococcus pneumoniae and Haemophilus influenzae, and the most common viruses were rhinovirus and adenovirus. The detection rate of Mycoplasma pneumoniae was 64.06%, and mixed infection rate was 50.47% (predominantly bacteria-virus co-infection). With increasing age, single bacterial/viral infection rates decreased, while M. pneumoniae and multi-pathogen mixed infection rates increased. Univariate analysis showed significant differences in initial body temperature, hospital stay, inflammatory indices (fibrinogen, CRP, D-dimer), immune ratios (NLR, PLR, SII) and pathogen type between sCAP and non-sCAP groups (all p5<0.05). Multivariate regression confirmed the initial body temperature, D-dimer, mixed infection and Mycoplasma. pneumoniae infection were independent risk factors for sCAP.

**Conclusion:**

Initial body temperature, D-dimer, mixed infection and M. pneumoniae infection are reliable predictors of pediatric sCAP. The constructed early prediction model has significant value for clinical diagnosis, treatment and prognosis evaluation of pediatric CAP.

## Background

Community-acquired pneumonia (CAP) is defined as an infectious inflammatory disease of the lungs caused by a confirmed pathogen, which occurs either in the outpatient setting or within the incubation period after hospital admission. Children represent a highly susceptible population to CAP (Torres et al., 2021; Rothberg, 2022). Globally, approximately 120 million new cases of CAP are reported annually, with nearly 1 million deaths occurring in children under 5 years of age (McAllister et al., 2019; Pratt et al., 2022). CAP is a major cause of childhood morbidity in developed countries and a leading contributor to childhood morbidity and mortality in developing countries (Qin and Shen, 2015). Therefore, the accurate identification and assessment of disease severity in CAP patients are of crucial importance for determining the appropriate treatment setting, rational use of antimicrobial agents, and reducing the economic burden on both individual patients and society as a whole.

Currently, there is no unified clinical assessment tool or consensus for evaluating CAP severity in clinical practice. The modified British Thoracic Society Pneumonia Score which includes confusion, uremia, respiratory rate, blood pressure, and age ≥ 65 years) is one of the most commonly used assessment tools in clinical settings (Ilg et al., 2019; Bradley et al., 2022). However, due to the lack of support from clinical biochemical indicators and etiological data, this score fails to reflect the degree of inflammation and infection in the patient’s body. Consequently, the clinical value of using the CURB-65 score alone for assessing the severity of CAP in patients is limited (Ilg et al., 2019).

In this study, we aim to analyze the composition of the etiological spectrum and the epidemiological characteristics of pathogens in hospitalized children with CAP. Additionally, we will investigate the clinical data of children with severe CAP to identify the characteristic factors associated with disease progression to severe status. Meanwhile, we will construct and validate a nomogram model. The overall objective of this research is to provide a scientific basis for the early identification of severe pneumonia, thereby offering valuable references for the clinical diagnosis and treatment of this disease.

## Methods

### Data source and inclusion criteria

Data on pediatric patients admitted to Jinan Children’s Hospital from January 2023 to December 2024 with a discharge diagnosis including community-acquired pneumonia (CAP) were collected from the Ruimei Laboratory Information System (LIS) platform. These data were used to analyze the potential influencing factors of severe and non-severe pneumonia, including the following variables: gender and age of the children; first body temperature during hospitalization, laboratory tests, pathogens, and length of hospital stay; and discharge diagnosis. Patients were excluded met the following criteria: incomplete clinical data; discharge diagnosis involving hospital-acquired pneumonia; immunocompromised status (including congenital immunodeficiency diseases, HIV infection, malignant tumors, receipt of anti-tumor or immunosuppressive therapy, and post-transplantation status); and non-infectious pneumonia. The diagnostic criteria for severe CAP (sCAP) were strictly based on the Clinical Guidelines for Community−Acquired Pneumonia in Children (2019 Version). A patient was diagnosed with sCAP if they met any of the following criteria: (1) poor general condition; (2) disturbance of consciousness, cyanosis, tachypnea (respiratory rate [RR] ≥70 breaths/min in infants, RR ≥50 breaths/min in children aged >1 year); (3) hypoxemia (accessory respiratory movements, intermittent apnea, or oxygen saturation <92%); (4) ultra−high fever or persistent high fever >5 days; (5) signs of dehydration or refusal to eat; (6) chest imaging showing ≥2/3 lung infiltration, multi−lobar involvement, pleural effusion, pneumothorax, atelectasis, lung necrosis, or lung abscess; (7) presence of extrapulmonary complications.

Etiological examinations were performed using nasopharyngeal swabs or sputum samples. All pathogens including bacteria, viruses, and Mycoplasma pneumoniae were detected by real−time fluorescent quantitative polymerase chain reaction (qPCR) following standard laboratory protocols to ensure consistency and reliability.

Feature selection and data preprocessing

Statistical analyses were performed using SPSS 26.0 software. Skewed continuous data were presented as median (interquartile range, IQR: Q1, Q3), and between-group comparisons were performed using the Mann-Whitney U test. Taking whether the discharge diagnosis was severe pneumonia as the outcome, multivariate Logistic regression analysis was performed. Cases with more than 20% of key clinical or laboratory indicators missing were excluded. For random missing data (<5% of key variables), multiple imputation was used; listwise deletion was applied only when missingness was completely random. Patients were randomly split into a training cohort (70%, 1040 cases) and a validation cohort (30%, 446 cases). Internal validation was performed using 5-fold cross-validation in the training set. A two-sided P < 0.05 was considered statistically significant. To minimize overfitting, multivariate logistic regression with forced entry was used; variables were selected based on clinical significance and univariate results (P < 0.1). Model stability was comprehensively evaluated using ROC curve, calibration curve, and Hosmer–Lemeshow test. Receiver operator characteristic (ROC) curves for risk prediction of children in the modeling group and validation group of the prediction model were plotted, and the area under the ROC curve (AUC) was calculated. Binary Logistic stepwise regression analysis was used to screen and identify risk factors for severe community-acquired pneumonia (CAP) in children, based on which a nomogram model was constructed. Meanwhile, the calibration degree of the model was evaluated using calibration curves, and the results of the Hosmer-Lemeshow goodness-of-fit test for the model were presented visually. A P < 0.05 was considered statistically significant.

## Results

### Epidemiology of pathogens

**Figure 1:**
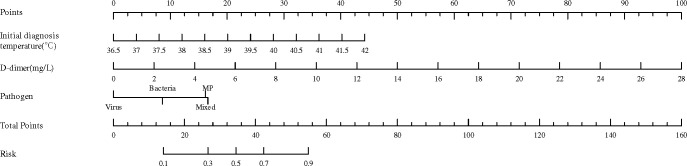
Nomogram for predicting the risk of sCAP. The probability of severe pneumonia can be directly calculated according to initial body temperature, D‑dimer and infection type.

A total of 1486 children with community - acquired pneumonia (CAP) who met the inclusion and exclusion criteria of this study were included in the analysis (see **Figure 1** for the flow chart of case inclusion and exclusion). There were 741 males and 745 females, with a male - to - female ratio of approximately 1:1. The age distribution was as follows: 47 cases aged 0–1 year, 149 cases aged 1–3 years, 680 cases aged 3–6 years, and 610 cases aged 6–18 years. Among these children, 920 had severe CAP and 566 had mild CAP.

The pathogen composition in this study is shown in **Table 1**. There were 736 cases of single-pathogen infection and 750 cases of multi - pathogen mixed infection in children with CAP. Among 952/1486 (64.06%) children infected with Mycoplasma pneumoniae, 490 cases (51.47%) were single Mycoplasma pneumoniae infection. Of the 338/1486 (22.75%) children with bacterial infection, 5 cases (1.48%) were single bacterial infection, mainly caused by Streptococcus pneumoniae and Haemophilus influenzae. For the 631/1486 (42.46%) children with viral infection, 12 cases (1.90%) were single viral infection. There were 185 cases of bacterial combined with viral infection, among which the combination of Streptococcus pneumoniae and rhinovirus was the most common (75 cases, 40.54%). There were 248 cases of bacterial combined with Mycoplasma pneumoniae infection, with the combination of Haemophilus influenzae and Mycoplasma pneumoniae being the most frequent (121 cases, 48.79%). There were 515 cases of viral combined with Mycoplasma pneumoniae infection, and the combination of rhinovirus and Mycoplasma pneumoniae was the most common (233 cases, 45.24%). Additionally, there were 54 cases of mixed infection with bacteria, virus, and Mycoplasma pneumoniae, among which the mixed infection of Haemophilus influenzae, rhinovirus, and Mycoplasma pneumoniae was the most prevalent (15 cases, 27.78%).

**Table 1:** Composition ratio of pathogens isolated from the CAP children (%).

Pathogen	Number of pathogens(n=2522)	Proportion(%)
**Mycoplasma**	**1345**	**53.33**
Drug-resistant mycoplasma	1159	45.96
Non resistant Mycoplasma	186	7.38
**bacteria**	**403**	**15.98**
Streptococcus pneumoniae	151	5.99
Haemophilus influenzae	157	6.23
Moraxella catarrhalis	34	1.35
Pseudomonas aeruginosa	4	0.16
Escherichia coli	2	0.08
Klebsiella pneumoniae	5	0.20
Serratia marcescens	1	0.04
Acinetobacter baumannii	13	0.52
Candida	10	0.40
Streptococcus intermedius	3	0.12
Bordetella pertussis	2	0.08
Grass green streptococcus	1	0.04
Staphylococcus aureus	10	0.40
Streptococcus pyogenes	5	0.20
Aspergillus flavus	1	0.04
Aspergillus fumigatus	1	0.04
Stenotrophomonas maltophilia	3	0.12
**virus**	**774**	**30.69**
epstein-barr virus	68	2.70
Rhinovirus	266	10.55
Parainfluenza virus	89	3.53
Bocavirus	17	0.67
Respiratory syncytial virus	59	2.34
Coxsackie virus	9	0.36
Adenovirus	110	4.36
Influenza a virus	56	2.22
Coronavirus	22	0.87
Metapneumovirus	42	1.67
Influenza B virus	15	0.59
Herpes simplex virus	11	0.44
Rotavirus	2	0.08
Cytomegalovirus	5	0.20
Norovirus	3	0.12

Bold numbers are used only to distinguish Mycoplasma, bacteria and virus subgroups and present the total case number and constituent ratio of each pathogen, without statistical significance.

**Figure 2:**
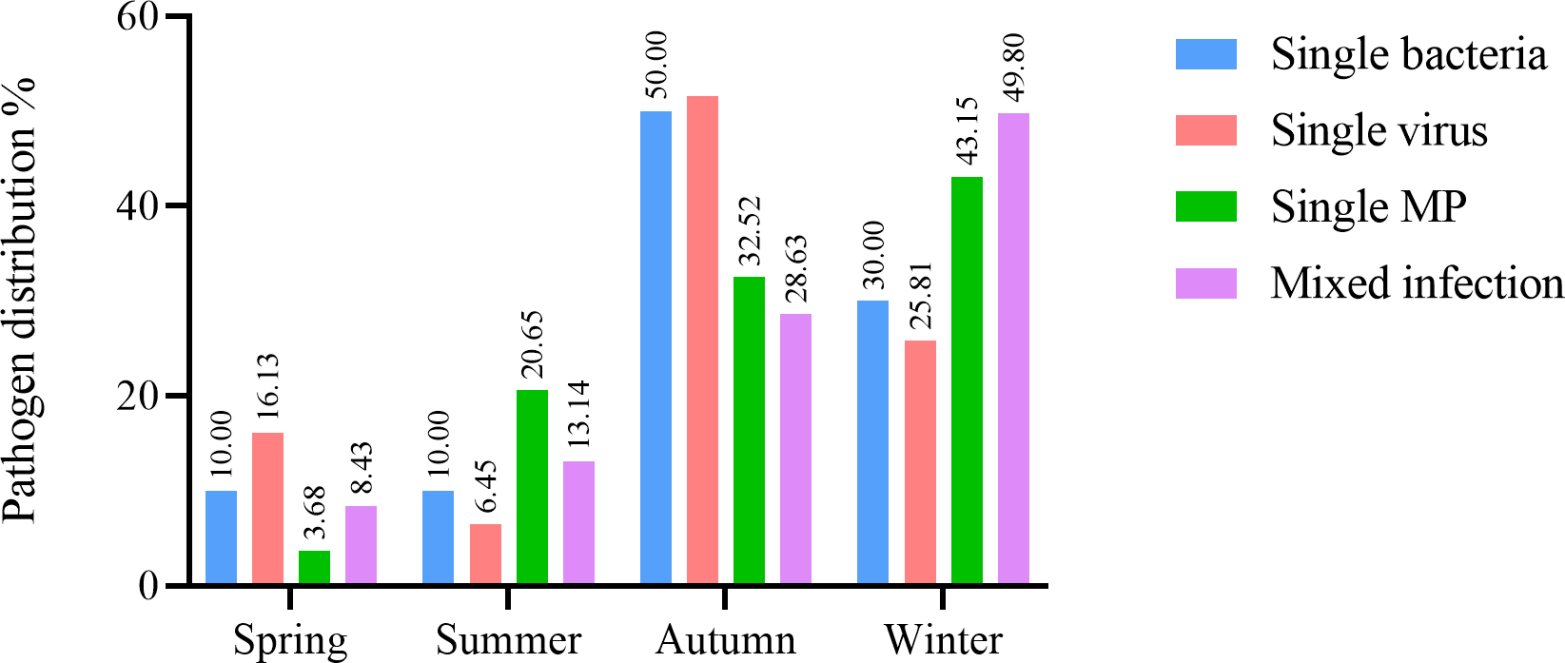
Pathogen distribution in CAP children in seasons.

**Figure 3:**
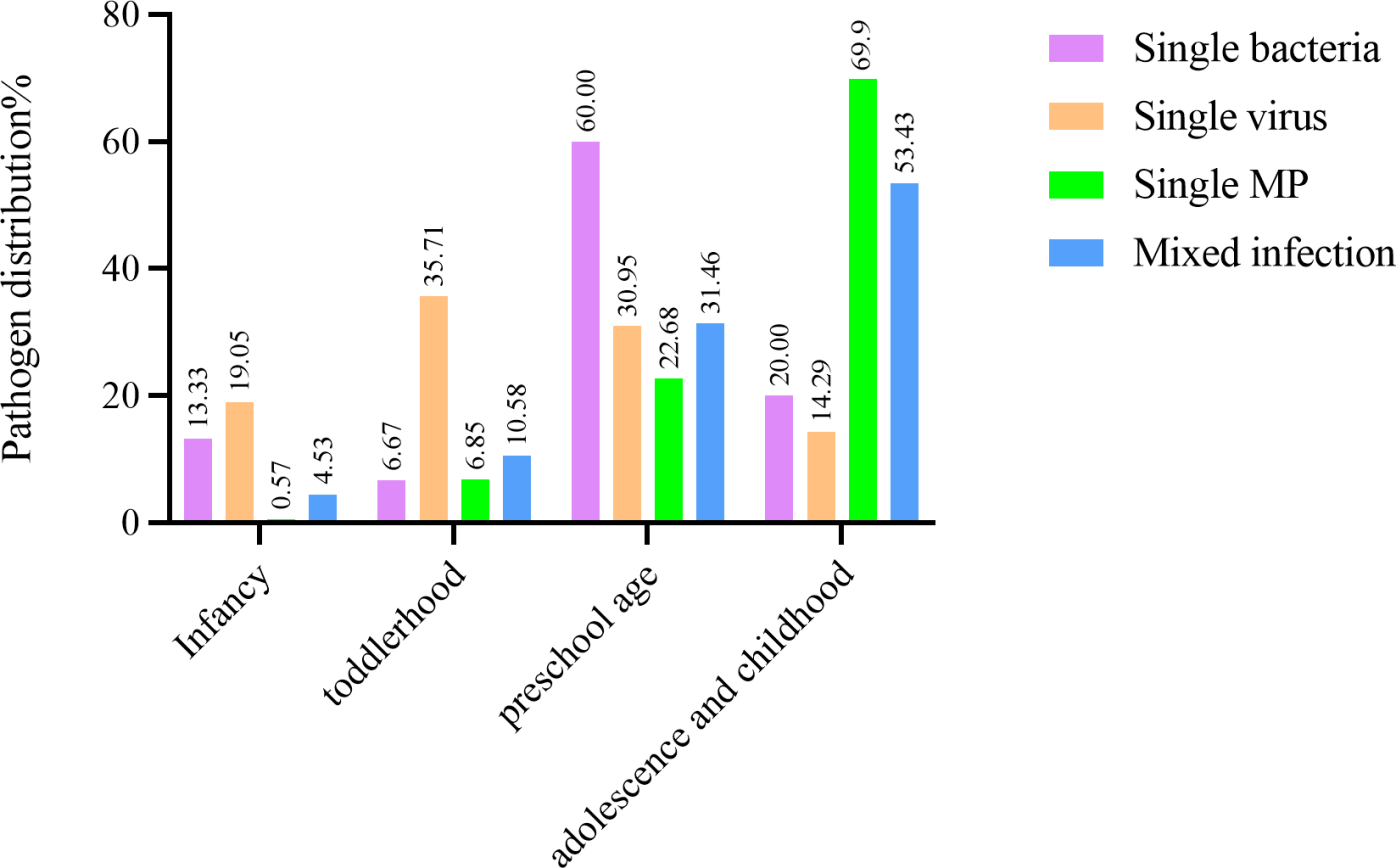
Pathogen distribution in CAP children of different ages.

**Figure 2** shows the pathogen composition in children of different ages in this study. With the increase of age, the proportion of single bacterial or viral infection showed a significant downward trend, while the proportion of Mycoplasma pneumoniae infection presented a marked upward trend. The proportion of multi - pathogen mixed infection accounted for 31.46% and 51.43% in the 3 - <6 years and 6 - <18 years age groups respectively, and this proportion also increased with age. As shown in **Figure 3**, the ratio of mild to severe CAP was approximately 1:1 across children of all age groups. In the 6 - <18 years age group, mild and severe cases accounted for 47.50% and 43.15% respectively, and the proportion increased with age.

**Figure 4:**
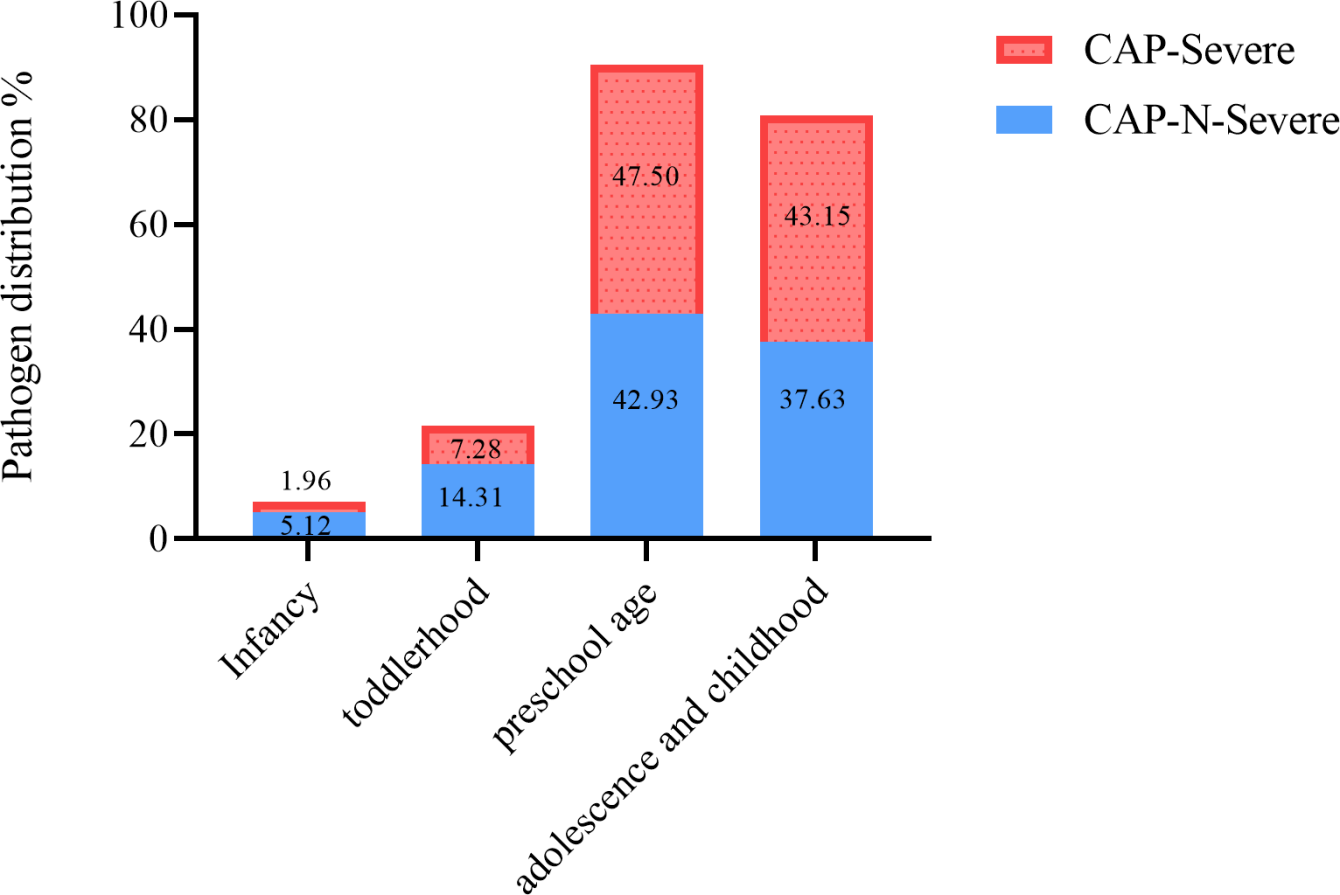
Distribution of mild and severe cases of CAP in children of different ages.

**Figure 4** shows the pathogen composition in pediatric CAP in different seasons. Single bacterial infection was most common in autumn; single viral infection was most frequent in spring; single Mycoplasma pneumoniae infection was most prevalent in winter, followed by autumn; and multi - pathogen mixed infection was most common in winter, followed by autumn.

### Establishment of cohort characteristic model

**Figure 5:**
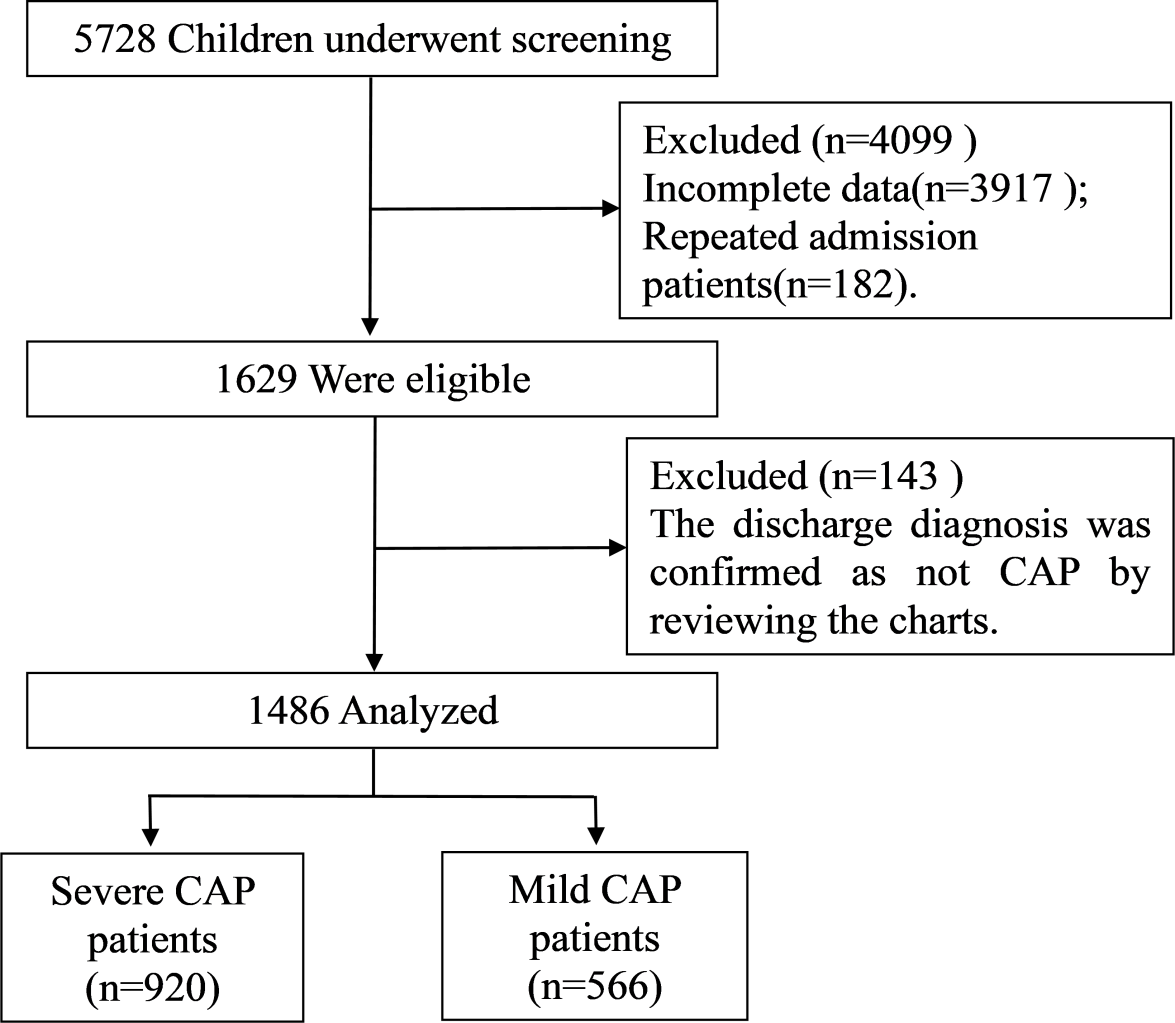
Nomogram for predicting the risk of sCAP. The probability of severe pneumonia can be directly calculated according to initial body temperature, D−dimer and infection type.

**Figure 6:**
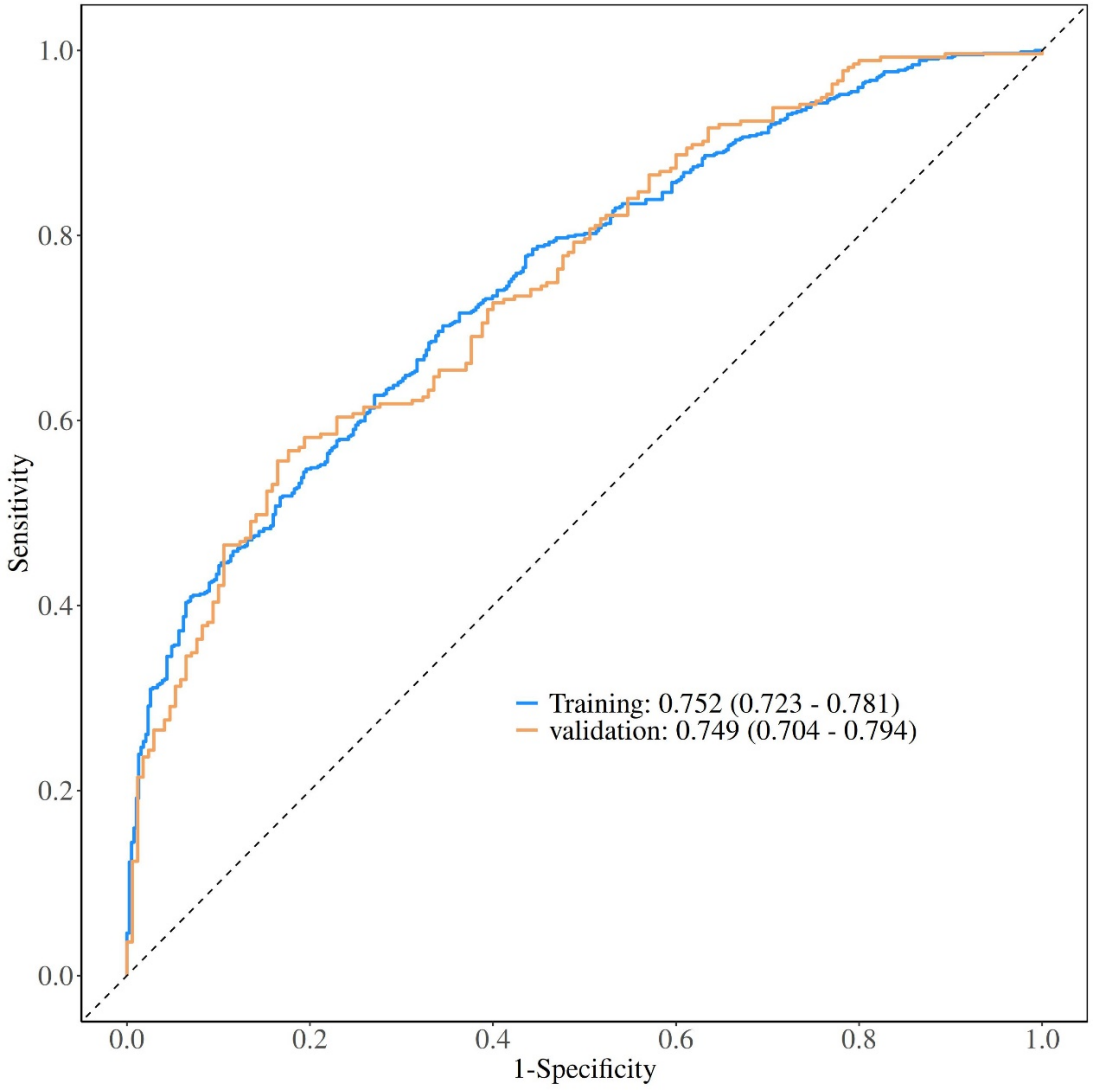
ROC curve of the prediction model. The AUC was 0.749, indicating favorable discriminative ability.

**Figure 7:**
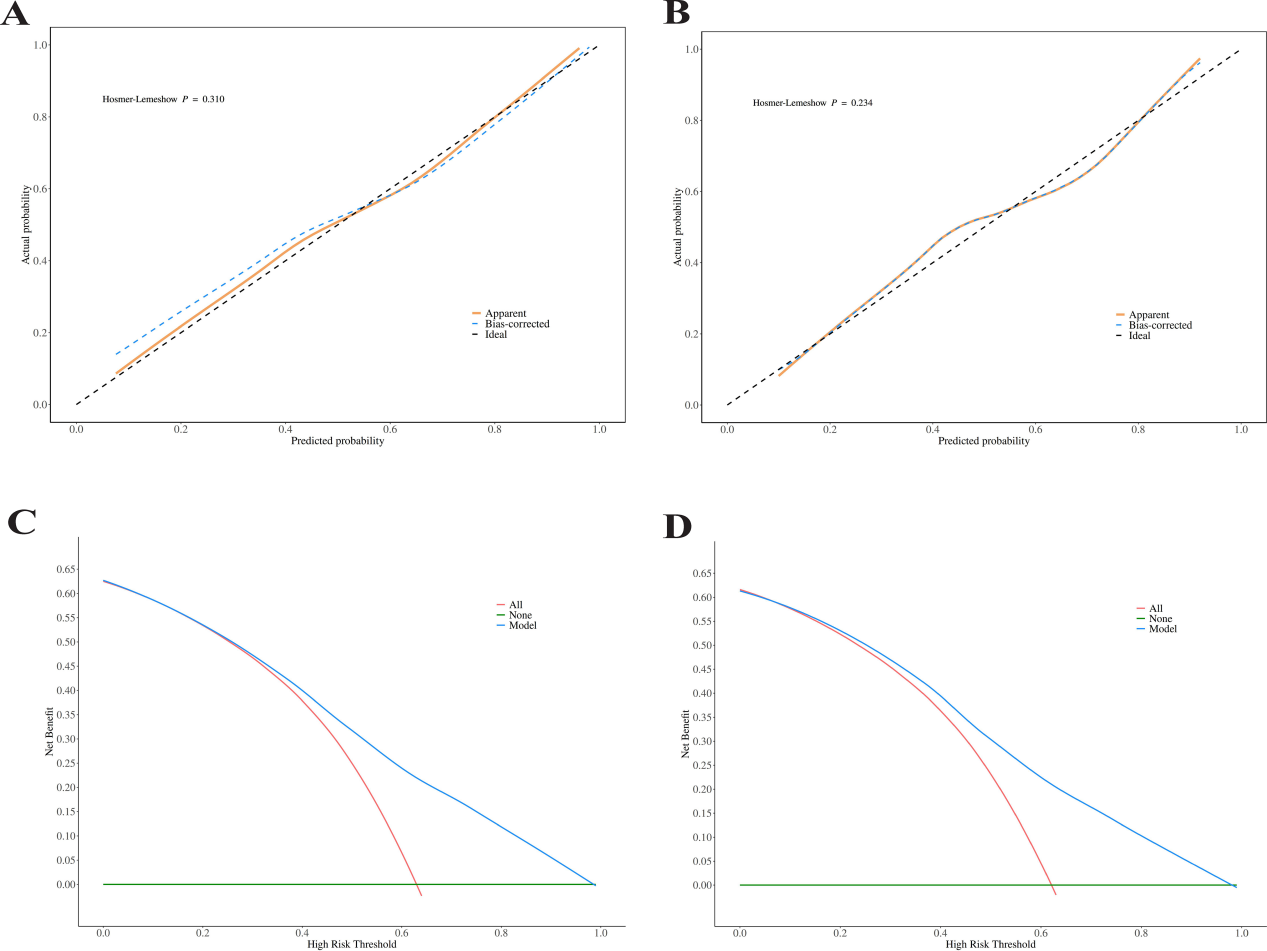
Calibration curve analysis and decision curve analysis of the nomogram model. **(A)** Calibration curve of the training set. **(B)** Calibration curve of the validation set. **(C)** Decision curve of the training set. **(D)** Decision curve of the validation set. The calibration curve was close to the reference diagonal line, suggesting good goodness of fit and reliable predictive accuracy.

Univariate analysis (**Table 2**) revealed significant between-group differences (P < 0.05) in the sCAP and non-sCAP groups in initial body temperature, hospital stay, fibrinogen, CRP, D−dimer, albumin, lymphocyte ratio, neutrophil ratio, NLR, PLR, SII, CALLY, and pathogen type. Continuous variables were compared by Mann-Whitney U test; categorical variables by χ² test. Multivariate Logistic regression confirmed that initial body temperature, D−dimer, mixed infection, and Mycoplasma pneumoniae infection were independent risk factors for sCAP in the modeling cohort. A nomogram model was constructed using thesefour variables (**Figure 5**). The model showed good discrimination, with an AUC of 0.749 (95%CI: 0.712–0.786) in the training cohort. (**Figure 6**). The Hosmer–Lemeshow test showed good model calibration (χ²=9.4008, df=8, P = 0.3096). Calibration curve showed high consistency between predicted and actual probabilities (**Figure 7**).

**Table 2:** Baseline characteristics of all the included patients.

Characteristic	CAP-N-severe (n = 566)	CAP-severe (n = 920)	Statistic	*P*
Sex, n(%)			χ²=6.45	**0.011**
Female	260 (45.94)	485 (52.72)		
Male	306 (54.06)	435 (47.28)		
Age(years), n(%)			χ²=37.89	**<.001**
0-1	29 (5.12)	18 (1.96)		
1-3	78 (13.78)	63 (6.85)		
3-6	163 (28.80)	247 (26.85)		
6-12	289 (51.06)	577 (62.72)		
12-18	7 (1.24)	15 (1.63)		
Pathogen, n(%)			χ²=33.48	**<.001**
Virus	33 (5.83)	9 (0.98)		
Bacteria	9 (1.59)	6 (0.65)		
MP	256 (45.23)	445 (48.37)		
Mixed	268 (47.35)	460 (50.00)		
Initial diagnosis temperature (°C)	39.00 (38.30, 39.40)	39.30 (39.00, 39.70)	Z=-11.88	**<.001**
Hospitalization days	6.00 (5.00, 7.00)	7.00 (6.00, 9.00)	Z=-11.93	**<.001**
FIB (g/L)	3.11 (2.65, 3.64)	3.29 (2.77, 3.82)	Z=-4.01	**<.001**
TC (mmol/L)	3.97 (3.53, 4.45)	3.87 (3.42, 4.42)	Z=-1.88	0.059
CRP (mg/L)	3.00 (3.00, 8.74)	5.84 (3.00, 15.21)	Z=-5.33	**<.001**
D-dimer (mg/L)	0.34 (0.24, 0.50)	0.55 (0.33, 1.20)	Z=-11.44	**<.001**
ALB (g/L)	40.90 (39.30, 42.30)	39.40 (37.10, 41.40)	Z=-9.94	**<.001**
WBC (◊10^9^/L)	9.29 (7.16, 12.08)	8.99 (6.98, 11.62)	Z=-1.55	0.122
LYM (%)	33.15 (24.02, 43.40)	27.35 (19.80, 37.20)	Z=-6.91	**<.001**
LYM (◊10^9^/L)	3.01 (2.07, 4.27)	2.45 (1.64, 3.48)	Z=-6.83	**<.001**
PA(mg/L)	161.00 (131.00, 198.00)	149.50 (116.00, 192.00)	Z=-4.28	**<.001**
PLT (◊10^9^/L)	348.00 (287.00, 432.75)	342.50 (271.00, 421.00)	Z=-1.66	0.097
NEU (%)	58.10 (46.90, 67.97)	64.00 (53.40, 72.30)	Z=-6.54	**<.001**
NEU (◊10^9^/L)	5.16 (3.62, 7.51)	5.49 (3.99, 7.61)	Z=-2.35	**0.019**
NLR	1.75 (1.09, 2.83)	2.34 (1.45, 3.66)	Z=-6.85	**<.001**
PLR	115.98 (86.40, 160.46)	136.25 (99.96, 194.69)	Z=-6.15	**<.001**
WLR	3.02 (2.30, 4.17)	3.66 (2.69, 5.06)	Z=-6.93	**<.001**
FAR	0.08 (0.06, 0.09)	0.08 (0.07, 0.10)	Z=-6.90	**<.001**
NPAR	1.45 (1.14, 1.69)	1.61 (1.34, 1.89)	Z=-8.97	**<.001**
NHANES	30.31 (11.65, 65.90)	16.32 (4.74, 48.79)	Z=-6.79	**<.001**
CLR	1.33 (0.61, 3.39)	2.46 (0.84, 8.03)	Z=-6.48	**<.001**
SII	603.79 (353.74, 1057.98)	751.63 (469.84, 1297.99)	Z=-5.62	**<.001**

FIB, fibrinogen; TC, Total Cholesterol; ALB, albumin; WBC, white blood cell; LYM, lymphocyte; PA, prealbumin; PLT, platelet; NEU, neutrophil; NLR, The ratio of neutrophils to lymphocytes; PLR, Ratio of platelets to lymphocytes; WLR, The ratio of white blood cells to lymphocytes; FAR, The ratio of fibrinogen to albumin; NPAR, Ratio of neutrophil count to albumin; NHANES, The product of albumin and lymphocytes divided by C-reactive protein; CLR, Ratio of C-reactive protein to lymphocytes; SII, The product of neutrophil and platelet counts divided by lymphocytes.

Bold values represent statistically significant difference at P < 0.01.

Univariate analysis of the baseline data of children with CAP (**Table 2**) showed that there were statistically significant differences (P < 0.05) between children with severe and non - severe CAP in terms of initial body temperature, length of hospital stay, fibrinogen, C - reactive protein (CRP), D - dimer, albumin, lymphocyte ratio, lymphocyte count, prealbumin, neutrophil ratio, neutrophil count, neutrophil - to - lymphocyte ratio (NLR), platelet - to - lymphocyte ratio (PLR), white blood cell - to - lymphocyte ratio (WLR), fibrinogen - to - albumin ratio (FAR), neutrophil - to - albumin ratio (NPAR), platelet count * neutrophil count/lymphocyte count (SII), albumin * lymphocyte count/(CRP * 10) (CALLY), and type of infectious pathogen. There were no statistically significant differences in the other indicators (P > 0.05).

The results of multivariate Logistic regression analysis of children with CAP showed that through the comparison of the balance of clinical data between the modeling group and the validation group (**Table 3**), with severe and non - severe conditions as dependent variables, the indicators with statistical significance in the univariate analysis were selected for binary Logistic regression collinearity analysis. The results indicated that initial body temperature, D - dimer, mixed infection, and Mycoplasma pneumoniae infection among the infection types were independent risk factors for severe community - acquired pneumonia in children in the modeling group.

**Table 3:** Comparison of laboratory markers between the training and validation sets.

Variables	CAP-Training set (n = 1040)	CAP-Validation set (n = 446)	Statistic	*P*
Initial diagnosis temperature(°C)	39.10 (38.60, 39.60)	39.00 (38.50, 39.50)	Z=-1.68	0.093
Hospitalization days	7.00 (6.00, 8.00)	7.00 (6.00, 8.00)	Z=-0.96	0.335
FIB(g/L)	3.19 (2.69, 3.74)	3.26 (2.79, 3.74)	Z=-1.52	0.130
TC(mmol/L)	3.95 (3.47, 4.43)	3.87 (3.45, 4.42)	Z=-1.15	0.249
CRP(mg/L)	4.73 (3.00, 12.10)	5.18 (3.00, 13.74)	Z=-0.84	0.399
D-dimer(mg/L)	0.43 (0.28, 0.88)	0.45 (0.29, 0.81)	Z=-0.24	0.808
ALB(g/L)	40.10 (38.10, 41.80)	40.10 (38.10, 41.70)	Z=-0.12	0.904
WBC(◊109/L)	9.13 (7.04, 11.75)	9.16 (7.02, 11.71)	Z=-0.19	0.847
LYM(%)	29.90 (20.90, 38.80)	29.35 (20.77, 40.10)	Z=-0.21	0.831
LYM(◊109/L)	2.63 (1.78, 3.80)	2.61 (1.76, 3.85)	Z=-0.09	0.927
PA(mg/L)	153.00 (123.00, 193.00)	153.00 (118.25, 197.75)	Z=-0.45	0.655
PLT(◊109/L)	347.00 (275.00, 425.00)	345.00 (279.00, 425.00)	Z=-0.32	0.747
NEU(%)	61.50 (51.50, 70.62)	62.30 (50.70, 71.20)	Z=-0.22	0.829
NEU(◊109/L)	5.42 (3.81, 7.52)	5.30 (3.84, 7.74)	Z=-0.06	0.950
NLR	2.05 (1.33, 3.34)	2.15 (1.26, 3.47)	Z=-0.21	0.830
PLR	128.57 (93.17, 181.95)	130.03 (91.75, 181.57)	Z=-0.14	0.885
WLR	3.34 (2.57, 4.78)	3.41 (2.49, 4.81)	Z=-0.21	0.831
FAR	0.08 (0.07, 0.09)	0.08 (0.07, 0.10)	Z=-1.53	0.126
NPAR	1.54 (1.27, 1.82)	1.54 (1.24, 1.81)	Z=-0.27	0.784
NHANE	21.34 (6.40, 56.75)	20.03 (5.74, 56.53)	Z=-0.57	0.567
CLR	1.85 (0.71, 5.94)	1.97 (0.70, 6.84)	Z=-0.51	0.612
SII	701.58 (431.74, 1186.42)	661.64 (414.57, 1188.44)	Z=-0.32	0.748

FIB, fibrinogen; TC, Total Cholesterol; ALB, albumin; WBC, white blood cell; LYM, lymphocyte; PA, prealbumin; PLT, platelet; NEU, neutrophil; NLR, The ratio of neutrophils to lymphocytes; PLR, Ratio of platelets to lymphocytes; WLR, The ratio of white blood cells to lymphocytes; FAR, The ratio of fibrinogen to albumin; NPAR, Ratio of neutrophil count to albumin; NHANES, The product of albumin and lymphocytes divided by C-reactive protein; CLR, Ratio of C-reactive protein to lymphocytes; SII, The product of neutrophil and platelet counts divided by lymphocytes.

A nomogram model for identifying children with severe CAP was constructed and validated based on the screening results of Logistic regression analysis. Three independent variables were included in the nomogram model, namely initial body temperature, D - dimer, and infection type (**Figure 5**). Through the nomogram, each variable value could be corresponding to a score, and the total score of all variables could be calculated, which was vertically corresponding to the predicted probability of severe CAP in children. The higher the total score, the higher the risk probability of severe illness.

The validation of the model mainly included discrimination and calibration. The receiver operating characteristic (ROC) curve was plotted (**Figure 6**) to evaluate the discrimination of the model, and the point with the maximum Youden index was taken as the optimal cut - off point. The specificity of the prediction model was 0.702, the sensitivity was 0.655, and the negative predictive value was 0.774, indicating that the model had good discriminative ability (**Table 4**). Meanwhile, the Hosmer - Lemeshow goodness - of - fit test showed that the model had a good fit (χ² = 9.4008, P = 0.3096), and the calibration curve also indicated that the predicted probability of the model was basically consistent with the actual probability, showing good calibration (**Figure 7**).

**Table 4:** The value of column chart model in identifying critically ill children with CAP.

Data	AUC (95%CI)	Accuracy (95%CI)	Sensitivity (95%CI)	Specificity (95%CI)	PPV (95%CI)	NPV (95%CI)	Cut off
Train	0.752 (0.723-0.781)	0.685 (0.655-0.713)	0.655 (0.607 - 0.702)	0.702 (0.667 - 0.738)	0.567 (0.521 - 0.613)	0.774 (0.740 - 0.807)	0.602
Test	0.749 (0.704-0.794)	0.652 (0.605-0.696)	0.624 (0.551 - 0.696)	0.669 (0.613 - 0.725)	0.538 (0.468 - 0.608)	0.742 (0.687 - 0.796)	0.602

CAP stands for community-acquired pneumonia; AUC is the area under the working characteristic curve of the subject; 95%CI is 95% confidence interval; PPV is positive likelihood ratio; NPV is Negative likelihood ratio.

## Discussion

Children are a high-risk population for community-acquired pneumonia (CAP) due to factors such as immature immune function and underdeveloped respiratory systems (Perin et al., 2022). Additionally, studies have reported that a large number of children with low-risk CAP are hospitalized for treatment, resulting in unnecessary economic expenditures (Jain et al., 2015; Weycker et al., 2021). Previous research has demonstrated that the disease burden of CAP differs significantly across regions, age groups, and pathogen types (Jiang et al., 2021; Ma et al., 2023).

In the present study, Mycoplasma pneumoniae (M. pneumoniae) was the most common pathogen causing CAP in hospitalized children, with a positive rate of 64.06%. This finding indicates that M. pneumoniae was the predominant pathogen in children with CAP admitted to our hospital during the two-year period from 2023 to 2024. The infection rate of pathogens exhibits significant regional and temporal variations, and infection rates vary substantially across studies. Furthermore, Previous studies has indicated that Mycoplasma pneumoniae infections typically reach an epidemic peak every 3–4 years (Eun et al., 2008). However, it remains unclear whether the relatively high detection rate of Mycoplasma pneumoniae in this study is associated with the occurrence of such an epidemic peak.

Among the bacteria detected in this study, Streptococcus pneumoniae and Haemophilus influenzae ranked as the top two. This is consistent with the 2019 version of the guidelines, which identify Streptococcus pneumoniae as the most common bacterial pathogen causing CAP in children. Regarding viral pathogens, rhinovirus and adenovirus were the most frequently detected in this study. This observation may be attributed to the fact that rhinovirus commonly colonizes the upper respiratory tract, while adenovirus is easily transmitted among children via droplets and contact.

Moreover, compared with children with non-severe CAP, those with severe CAP had longer hospital stays, higher hospitalization costs, and a higher incidence of adverse outcomes.

For clinicians, the key to reducing mortality and complications, as well as rationalizing the allocation of medical resources and alleviating the economic burden on patients, lies in the timely and accurate identification and assessment of the severity of illness in pediatric patients. Currently, no unified diagnostic criteria exist for severe pediatric community-acquired pneumonia (sCAP). The evaluation is solely based on clinical symptoms and signs, without integration of auxiliary examinations, which consequently increases the rate of missed diagnosis. Thus, there is an urgent need to develop a scoring model capable of effectively identifying children with severe CAP, so as to improve survival rates and achieve the goal of precision treatment.

Against this backdrop, the present study screened for potential risk factors associated with severe community-acquired pneumonia (sCAP) in children. The results showed that initial body temperature, hospital stay, fibrinogen, CRP, D-dimer, albumin, prealbumin, lymphocyte and neutrophil indicators, multiple immune-inflammatory ratios (NLR, PLR, WLR, FAR, NPAR, SII, CALLY), and pathogen type were significantly different between the sCAP and non-sCAP groups (all P < 0.05). These variables could serve as potential predictors of severe CAP.

Subsequently, the variables with statistically significant differences were incorporated into a binary multivariate Logistic regression analysis. The results indicated that initial body temperature, D-dimer levels, and infectious types (including mixed infections and Mycoplasma infections) were independent risk factors for severe CAP in the modeling cohort of pediatric patients. D-dimer is a biomarker of endothelial injury, hypercoagulability, and microthrombosis. Elevated D-dimer indicates activation of coagulation and fibrinolysis, which is closely associated with severe inflammation and disease progression. Mixed infection exacerbates the inflammatory response, impairs therapeutic efficacy, and increases the risk of severe CAP. These biological mechanisms support the rationality of the model.

Body temperature refers to the body’s core temperature, is maintained relatively constant through the body’s intrinsic regulatory mechanisms. It undergoes varying degrees of alteration in the context of disease (Cramer et al., 2022). Therefore, measuring the body temperature of pediatric patients at the time of hospital admission can be used as an inflammatory indicator for monitoring the disease progression of CAP in children.

D-dimer is a degradation product of cross-linked fibrin clots generated by plasmin-mediated fibrinolysis, and thus reflects the fibrinolytic activity of the body. Elevated D-dimer levels often indicate a hypercoagulable state and secondary fibrinolytic hyperactivity. Previous studies (He et al., 2020; Zhang et al., 2024) have demonstrated that D-dimer holds clinical significance in the assessment of severe pneumonia. Additionally, research (Zhu et al., 2019; Li, 2019; Zhang and Wang, 2020; Li et al., 2023) has shown that children with Mycoplasma pneumoniae pneumonia frequently exhibit elevated plasma D-dimer levels and a hypercoagulable state. This phenomenon is attributed to the activation of inflammatory mediators following Mycoplasma pneumoniae infection, which causes damage to capillary endothelial tissue. The subsequent release of procoagulant substances disrupts the normal function of the coagulation system, leading to the formation of microthrombi, and in severe cases, pulmonary embolism, which ultimately results in lung tissue necrosis.

In recent years, abnormalities in coagulation function have garnered increasing attention from clinicians. Elevated plasma D-dimer levels can, to a certain extent, reflect the severity of severe pneumonia. Early anticoagulant therapy in these children may reduce mortality and improve prognosis (Huang et al., 2021; Wang and Tian, 2023). In summary, pediatric patients with severe CAP have a potential risk of developing disseminated intravascular coagulation (DIC). Given the lack of specific clinical manifestations during this stage, the inclusion of D-dimer in the predictive model can alert clinicians to the progression of CAP in children.

Mixed infections may be the primary reason for the poor therapeutic response to monotherapy in some pediatric CAP patients, while also increasing the difficulty of treating CAP. In recent years, Mycoplasma infection has gradually emerged as a key pathogen causing pneumonia in children, with a distinct seasonal pattern. It is most commonly observed in children aged 6–12 years, and its incidence increases with age. This trend may be associated with the close contact among children, which facilitates the transmission of the pathogen. The incorporation of these two infectious pathogen types into the model significantly enhances the model’s credibility.

Subsequently, in this study, a predictive model for severe community-acquired pneumonia (CAP) was constructed in the modeling group, and a receiver operating characteristic (ROC) curve was plotted. Based on this, the optimal predictive probability was calculated, which was then used for validation in the validation group. The validation results showed that the specificity of this predictive model was 0.702, the sensitivity was 0.655, and the area under the ROC curve (AUC) was 0.749. These findings indicate that the model has showed moderate but clinically useful predictive performance for severe pediatric CAP.the risk of severe CAP. Meanwhile, based on the results of binary multivariate Logistic regression analysis, this study identified meaningful variables, including the initial body temperature, D-dimer, and infection type, as the variables for the nomogram model. The nomogram model was presented in a visualized form, which enhanced the readability of the results. Furthermore, the model performance evaluation results demonstrated that the calibration curve had a good fit with the standard curve, and the Hosmer-Lemeshow test confirmed a high degree of consistency, suggesting a high prediction accuracy of the model.

This study has several limitations. First, this is a single-center retrospective study, which mayintroduce selection bias, information bias, and confounding, thereby limiting external validity. Second, the model was validated internally only; external validation using multicenter cohorts is needed to improve generalizability. Third, due to the retrospective design, some confounders including vaccination status, prior antibiotic use, and detailed nutritional status were not fully collected. Fourth, the study focused on severity at admission and did not evaluate long-term outcomes such as ICU admission, complications, or recurrence. Despite these limitations, the model shows good predictive performance and clinical value. Future prospective, multicenter studies with larger samples and complete confounder data are warranted to further validate and optimize the model.

## Data Availability

The data analyzed in this study is subject to the following licenses/restrictions: The data that support the findings of this study are available from the corresponding author upon reasonable request. Requests to access these datasets should be directed to Xin Lv, etyyjyklvxin@163.com.
